# Quorum quenching, lactic acid bacteria and microencapsulation as emerging and promising strategies to mitigate tomato soft rot

**DOI:** 10.3389/fpls.2026.1812455

**Published:** 2026-07-06

**Authors:** Botagoz Mutaliyeva, Tajalli Keshavarz, Godfrey Kyazze, Marko Vinceković, Galiya Madybekova, Assem Issayeva, Ussen Akhanov

**Affiliations:** 1 Department of Biotechnology, M.Auezov South Kazakhstan University, Shymkent, Kazakhstan; 2 School of Life Sciences, University of Westminster, London, United Kingdom; 3 Department of Chemistry, Division of Agroecology, Faculty of Agriculture, University of Zagreb, Zagreb, Croatia; 4 Department of Chemistry, O. Zhanibekov South-Kazakhstan Pedagogical University, Shymkent, Kazakhstan; 5 Scientific and Production Enterprise “Antigen” LLP, Abai, Almaty, Kazakhstan; 6 Department of Materials Science, Nanomaterials and Engineering Physics, K. Satbayev Kazakh National Research Technical University, Almaty, Kazakhstan

**Keywords:** lactic acid bacteria, microencapsulation, mitigation strategies, quorum quenching, quorum sensing, soft rot bacteria, tomato

## Abstract

Bacterial soft rot caused by *Pectobacterium* spp. and *Dickeya spp*, represents a major challenge in tomato production, leading to significant pre- and postharvest losses worldwide. Conventional control strategies are largely based on physical, environmental and chemical measures; however, their effectiveness is often limited by resistance development, environmental concerns, and increasing regulatory restrictions. This review critically examines current and emerging strategies to mitigate tomato soft rot, including biological control, advanced targeted and integrated technologies. While certain strategies (RNAi, UV-C, nanotechnology, and breeding) are discussed concisely, particular attention is given to quorum quenching (QQ) as an anti-virulence strategy targeting bacterial communication systems (quorum sensing), as well as to lactic acid bacteria (LAB) and formulation technologies as the most promising directions. Recent literature indicates that LAB have been evaluated in a moderate number of studies, including numerous *in vitro* assays and a smaller set of *in vivo* experiments on detached fruits and in greenhouse systems, where they exhibit antagonistic activity against soft rot pathogens. Reported mechanisms include the production of antimicrobial metabolites, competitive exclusion, and quorum sensing interference. However, existing reviews tend to address these strategies separately, while field-scale evidence and the integration of practical delivery systems remain limited. This review provides a focused and integrative perspective on the combined application of QQ, LAB, and microencapsulation technologies for the mitigation of tomato soft rot. Special attention is given to the emerging role of LAB as potential quorum quenching agents, the limitations associated with their stability and field performance, and the potential of microencapsulation to enhance their viability, enable controlled release, and improve overall efficacy. The proposed integrative framework offers new insights into the development of sustainable and application-oriented biocontrol solutions for soft rot pathogens.

## 1 Introduction

Soft rot is a disease affecting many agricultural and horticultural crops, mainly caused by members of genres pectobacteriaceae and Dickeyya, which are significant threats to tomato cultivation (Solanum lycopersicum) worldwide. As being a soft fruit with high water content, Tomatoes are especially vulnerable to this disease (Ahmed et al., 2017b). These pathogens rely on quorum sensing to regulate the production of virulence factors, including cell-wall-degrading enzymes, such as pectinases, cellulases, and proteases, that degrade plant tissue (Stevens et al., 2004; Jeyakumar et al., 2024). The optimal temperature range for the growth of soft rot-causing bacteria is approximately 16–34 °C, and disease development is favored under conditions of high humidity and warm temperatures. These factors contribute to significant postharvest losses, estimated at approximately 15–30% of harvested tomatoes, with values reaching up to 50% under severe conditions, across regions with varying levels of economic development, and up to 60% in the developing countries (Rai et al., 2022; Jeyakumar et al., 2024; Mengistu and Sharew, 2024; Felicioni et al., 2025; Oladipo et al., 2026).

These estimates primarily refer to postharvest stages, including storage and transportation, primarily due to microbial decay, including tomato soft rot, where environmental conditions promote pathogen proliferation (Rai et al., 2022; Mengistu and Sharew, 2024).

According to (Kolomiiets et al., 2020), the impact of these pathogens extends beyond the immediate losses of crops; they negatively affect the general health of the plant, which leads to a vigor reduction and greater susceptibility to secondary infections (Kolomiiets et al., 2020).

Soft rot has an economic and agricultural impact on global production in the world, and cause losses during all stages: cultivation, post-harvest storage and transportation (Singh et al., 2017; Zhang et al., 2021; Gautam et al., 2024). In addition to economic losses, soft rot undermines the quality and marketability of tomatoes, presenting challenges for both small-scale farmers and commercial growers. This continuous threat necessitates integrated disease management strategies that encompass all growth and post-harvest stages.

The importance of soft rot in tomato plants requires urgent attention to develop and implement effective mitigation strategies. Historically, control strategies for soft rot have relied on prevention and included measures such as field hygiene, crop rotation, which interrupt the life cycles of pathogens, adequate drainage and monitoring moisture levels, use of chemical treatments to suppress bacterial populations, irrigation practices and that are essential to healthiest growth of plants and reducing the prevalence of the disease (Mola et al., 2024). It has been observed that excessive humidity and poor air circulation around plants create ideal conditions for the pathogens of soft rot.

However, using chemical treatments to prevent this disease causes new challenges such as pesticide resistance and environmental pollution (Harsimran Kaur and Harsh, 2014).

In conjunction with preventive and cultural strategies, biological control, such as beneficial microorganisms and bacteriophages, provides an eco-friendly alternative to chemical treatments. This is an emerging strategy that takes advantage of natural antagonists to suppress pathogens of soft rot. The application of microbial agents, such as *Bacillus* spp. and *Trichoderma* spp., was documented to give protective advantages to tomato plants, effectively reducing the seriousness of diseases and promoting healthier growth.

Another biocontrol approaches involves the use of plant-derived extracts, such as those from oleander, chili, mint, garlic, turmeric, and neem, which have been shown to inhibit soft rot pathogens and may induce systemic resistance in tomato plants against subsequent infections (Akbar Khan et al., 2014).

Although more research is needed to optimize the application of biological control agents in field situations, preliminary results are encouraging and point to an integrated disease management approach.

Recent advances in the technologies that offer promising approaches to mitigate soft rot such as the development of resistant tomato cultivars through conventional breeding and genetic engineering, hold potential for long-term environmentally friendly alternative to disease management and sustainability in tomato production. While emerging technologies, such as nanotechnology (Gautam et al., 2024), RNA interference (RNAi) (Xiong et al., 2005; Wang et al., 2017; Ali et al., 2024) UV-C-based postharvest treatments (Stevens et al., 2004), and plant-derived compound (Qianqian et al., 2024), also offer approaches to combat soft rot effectively, this review focuses on quorum sensing-regulated virulence in *Pectobacterium* and *Dickeya* and critically evaluates biological strategies, with emphasis on lactic acid bacteria (LAB), and formulation technologies, including microencapsulation, as sustainable tools to manage tomato soft rot.

Although several recent reviews have addressed the biology of soft rot pathogens and summarized existing disease management approaches (Perfileva et al., 2025), including quorum quenching-based biocontrol strategies (Zhu et al., 2023), and LAB-mediated plant disease control (Raman et al., 2022), limited attention has been given to the integrated application of quorum quenching approaches, lactic acid bacteria (LAB), and microencapsulation technologies for sustainable mitigation of tomato soft rot.

In this review, we provide a focused and integrative perspective on the combined application of QQ, LAB, and microencapsulation technologies for sustainable soft rot mitigation. Particular attention is given to the emerging role of LAB as potential quorum quenching agents, the challenges associated with their environmental stability and field performance, and the potential of microencapsulation systems to enhance bacterial viability, enable controlled release, and improve overall biocontrol efficacy. Furthermore, this review highlights application-oriented and environmentally sustainable strategies that may contribute to the development of next-generation soft rot management approaches.

Consequently, addressing the challenges posed by soft rot pathogens requires collaborative efforts among researchers, farmers, and agricultural stakeholders. The integration of preventive measures, innovative cultural practices, biological control strategies, quorum quenching approaches, and resistant plant varieties may provide a synergistic framework for sustainable disease management. Such multifaceted strategies not only improve crop health and productivity but also support environmentally sustainable agricultural practices capable of adapting to the challenges associated with plant disease management under changing climatic conditions (Akbar Khan et al., 2015).

By examining pathogen biology, current control measures, and emerging integrative approaches involving quorum quenching, LAB, and microencapsulation technologies, this review aims to support researchers, and agricultural practitioners in developing sustainable and application-oriented solutions for the effective management of tomato soft rot.

## 2 Phytopathogenic bacteria involved in soft rot: biology, virulence mechanisms, and relevance to tomato

### 2.1 Pathogens: *Pectobacterium* and *Dickeya*

Soft rot in tomatoes is caused primarily by two bacterial genera: *Pectobacterium* (formerly *Erwinia*) (Farrar et al., 2000; Pérombelon, 2002), and *Dickeya* (formerly part of *Pectobacterium*) (Agyemang et al., 2020; Ge et al., 2021). Both are Gram-negative, facultatively anaerobic bacteria belonging to the family Enterobacteriaceae. Initially, according to the taxonomic classification of these pathogens, soft rot phytopathogens were identified as *Bacillus carotovorus* (Lacey, 1926; Motyka et al., 2017*)*. Subsequently, they were included in the genus *Erwinia* as two separate species: *Erwinia carotovora* and *Erwinia chrysanthemi* (Burkholder et al., 1953), then they were transferred to the genus *Pectobacterium* as *Pectobacterium carotovorum* and *Pectobacterium chrysanthemi*, respectively (Hauben et al., 1998). There is some literature with descriptions of the genus *Pectobacterium*, including seven species listed in **Table 1**.

**Table 1:** Classification of the genus Pectobacterium supplemented with host range, optimal temperature, and relevance to tomato.

Name of genus/species/subspecies	Host range	Optimal temperature, ^0^C	Relevance to tomato	References
*Pectobacterium carotovorum:* 4 subspecies	Wide (vegetables, ornamentals)	25-30	Major soft rot pathogens	(Duarte et al., 2004; Koh et al., 2012; Nabhan et al., 2012; Nabhan et al., 2013; Agyemang et al., 2020)
*Pectobacterium carotovorum subsp.brasiliense*	Potato, tomato, vegetables, Ornamental bulbous plant	25-30	One of the emerging soft rot pathogen of tomato	(Nabhan et al., 2013; Zhang et al., 2016; Li et al., 2019; Fei et al., 2026)
*Pectobacterium carotovorum subsp.odoriferum*	Vegetables (cabbage, slimy rot of witloof chicory	25-30	Limited evidence of occurrence	(Nabhan et al., 2013; Zhang et al., 2016; Li et al., 2019)
*Pectobacterium carotovorum subsp.carotovorum*	a wide range of host plant species (cabbage, tomatoes, carrots, onions and potatoes)	25-30	Major and primarily caused soft rot pathogens in tomato	(Duarte et al., 2004; Koh et al., 2012; Nabhan et al., 2012; Zhang et al., 2016; Li et al., 2019; Kolomiiets et al., 2020; Anandan and Vittal, 2025; Nam and Kim, 2026; Xie et al., 2026; Zhang et al., 2026b)
*Pectobacterium carotovorum* subsp. *actinidiae*	canker-like symptoms in yellow kiwifruit	The optimal temperature range for the growth of the isolated strains was 30–32 °C (disease occurs in the summer instead of early spring)	In association with tomato soft rot appears to be limited and poorly documented compared with major tomato soft rot pathogens such as Pectobacterium Brasiliense and Pectobacterium carotovorum subsp.carotovorum.	(Duarte et al., 2004; Koh et al., 2012; Nabhan et al., 2012; Zhang et al., 2016)
*Pectobacterium aroidearum* (Pectobacterium aroidearum sp. nov.)	preference for monocoty-ledonous plants	28-32 ^0^С Humidity and warm conditions	To date, evidence supporting a significant role of Pectobacterium aroidearum in tomato soft rot is scarce, whereas the bacterium is more frequently associated with soft rot diseases of monocotyledonous and various vegetable hosts (for example, taro, calla lily, Chinese cabbage, pepper, cxarrot, potato, zucchini, konjac)	(Nabhan et al., 2013; Zhang et al., 2016; Toth et al., 2021; Li et al., 2022)
*Pectobacterium atrosepticum*	Potato (cool climates)	18-25 ^0^С	One of the soft rot pathogen of tomato	(Gardan et al., 2003; Czajkowski et al., 2015; Zhang et al., 2016; Li et al., 2019; Agyemang et al., 2020; Kolomiiets et al., 2020)
*Pectobacterium wasabiae*	Potato, vegetables	20-30	Emerging relevance	(Gardan et al., 2003; Nabhan et al., 2013; Li et al., 2019)
*Pectobacterium parmentieri*	Potato	25-30	Potential emerging pathogen	(Khayi et al., 2016; Li et al., 2019)
*Pectobacterium betavasculorum*	exclusively on sugar beet	25-30 ^0^C	evidence of its involvement in tomato is lacking	(Gardan et al., 2003; Nedaienia and Fassihiani, 2011; Motyka et al., 2017)
*Pectobacterium cacticida*	Primarily associated with cactus and succulent plants, especially aspecies of Cactaceae. Its host range appears relatively narrow and specialized, in contrast to many others Pectobacterium species that infect a wide variety of vegetable crops.	25-30	There is currently no evidence supporting the involvement of Pectobacterium cacticida in tomato soft rot	(Hauben et al., 1998; Nabhan et al., 2013; Portier et al., 2019; Su et al., 2022; Jonca et al., 2024)

These pathogens are globally distributed and have been identified as major contributors to losses in tomato and other crop yields. Among the genus *Pectobacterium* species such as *Pectobacterium carotovorum* and *Pectobacterium atrosepticum*, are commonly associated with soft rot in a wide range of crops, including tomatoes (Agyemang et al., 2020; Gautam et al., 2024; Zhang et al., 2024) peppers (Tang et al., 2025). Some works related to the identification of soft rot pathogens in tomatoes confirmed that the causative agent is *P. carotovorum* subsp. *сarotovorum* (Kolomiiets et al., 2020). These bacteria thrive in warm, wet environments and are highly adaptable to diverse ecological conditions.

The genus Dickeya, formerly part of *Pectobacterium*, is described as a pathogen of tomato soft rot agents, including species such as *Dickeya dadantii* and *Dickeya dianthicola* (Samson et al., 2005; Parkinson et al., 2014*)* established the genus *Dickeya*, separating it from *Pectobacterium* and describing species such as *D. dadantii*. According to the generally accepted classification, eight *Dickeya* species are distinguished nowadays, as can be seen from **Table 2**.

**Table 2:** Classification of the genus *Dickeya* supplemented with host range, optimal temperature, and relevance to tomato.

Name of genus/species/subspecies	Host range	Optimal temperature, ^0^C	Relevance to tomato	References
*Dickeya aquatica*	Environment-associated *Dickeya*	Mesofilic conditions 25-30 ^0^C	There is currently no evidence supporting the involvement of Dickeya aquatica in tomato soft rot.	(Parkinson et al., 2014)
*Dickeya chrysanthemi*	Ornamentals	25-30	Occasional relevance	(Samson et al., 2005)
*Dickeya* *dadantii* (involving *D. dadantii* subsp.	Wide (vegetables, ornamentals, fruit crops)	28C-30C	Major soft rot pathogen causing tissue maceration in tomato	(Samson et al., 2005; Brady et al., 2012; Banerjee et al., 2022; Ghasemi Alaleh et al., 2025)
*Dickeya* *dianthicola*	Potato, ornamentals	25-30	Moderate relevance	(Samson et al., 2005; Toth et al., 2011)
*Dickeya fangzhongdai*	ornamental plants, monocots (for example, orchids), dicots (for example, *Pyrus pyrifolia)*	28-32 ^0^C Warm and humid conditions	No evidence/no documented	(Tian et al., 2016)
*Dickeya paradisiaca*	It is associated with banana, and tropical plants, where it causes soft rot and pseudostem rot. Reported on maize and some ornamental plants	28-34 ^0^C High humidity	No evidence/no documented	(Samson et al., 2005)
*Dickeya solani*	Primarily to potato, but also vegetables, ornamentals	18-37 Different climatic conditions Maximal activity at 28C, and can grow at more high temperature than P. Carotovorum	Moderate to high relevance to tomato due to similar soft rot mechanisms	(Toth et al., 2011; Dickeya solani sp. nov. a pectinolytic plant-pathogenic bacterium isolated from potato (Solanum tuberosum), 2014; Golanowska et al., 2017)
*Dickeya zeae*	Primarily monocots (maize, rice, banana), also potato, strain-dependent-variability	30-37 high temperature and humidity	Limited relevance	(Samson et al., 2005; Hu et al., 2022) Useful model for soft rot mechanisms (Hu et al., 2018)

These pathogens are considered more aggressive and are known to cause outbreaks under specific environmental conditions, often associated with higher temperatures and humid climates. Both genera are known for their opportunistic nature, infecting plants through wounds, natural openings such as stomata or lenticels, and areas weakened by stress or mechanical damage. They have an optimal temperature range for growth from 25 °C to 30 °C (Jeyakumar et al., 2024).

Thus, not all members of the genera *Pectobacterium* and *Dickeya* contribute equally to tomato soft rot, among *Pectobacterium, Pectobacterium brasiliense* and *Pectobacterium carotovorum subsp.carotovorum* are most frequently reported in tomato, whereas other species such as *Pectobacterium aroidearum, Pectobacterium betavasculorum, Pectobacterium cacticida* show limited or no documented relevance to tomato soft rot because of their other specific host. Similarly, within the genus Dickeya, the most major pathogens for tomato are Dickeya dadantii and Dickeya solani, while others such as Dickeya aquatica, Dickeya fangzhongdai, Dickeya paradisiaca are associated with environmental reservoirs or non-tomato hosts.

This species-level differentiation is critical, as virulence potential and ecological adaptation vary substantially within both genera. In tomato, disease development is strongly dependent on the regulation of plant cell-wall-degrading enzymes (PCWDEs), including pectinases, cellulases, and proteases.

Mechanism of disease includes the adhesion of bacteria to the plant surface, multiplication, and the formation of biofilms to protect them from environmental stresses and host defenses. Cell-degrading enzymes participate in the destruction of the plant tissues from small, water-soaked lesions, resulting to tissue maceration and plant death.

The most important virulence factors are signal molecules for bacterial communication, and when the threshold concentration level is achieved, bacteria reveal them and can react by expression of specific genes responsible for secrete of the cell-degrading enzymes as described in works (Dong et al., 2007; Wang et al., 2020*)*. Some pathogenic bacteria can produce virulence factors making them more harmful (Majumdar and Pal, 2016; Mukherjee and Bassler, 2019). The threshold concentration depends on bacterial population density: when the number of bacteria increases, the concentration of signal molecules increases (Sjöblom et al., 2006). This phenomenon is called quorum sensing, and understanding the mechanism of quorum sensing can help to develop strategies to prevent soft rot disease in plants. For example, preventing biofilm formation is also one of the strategies for the destruction of QS, because of the low concentration of bacteria. Some strategies take into account the knowledge about different types or categories of QS molecules based on bacteria type. One of the most well-known signaling molecules is N-acyl homoserine lactones, (AHLs), which regulate the transcription of genes responsible for key traits, including virulence and biofilm formation (March and Bentley, 2004; Sibanda et al., 2018; Vesuna and Nerurkar, 2020; Alymanesh et al., 2024).

**Figure 1:**
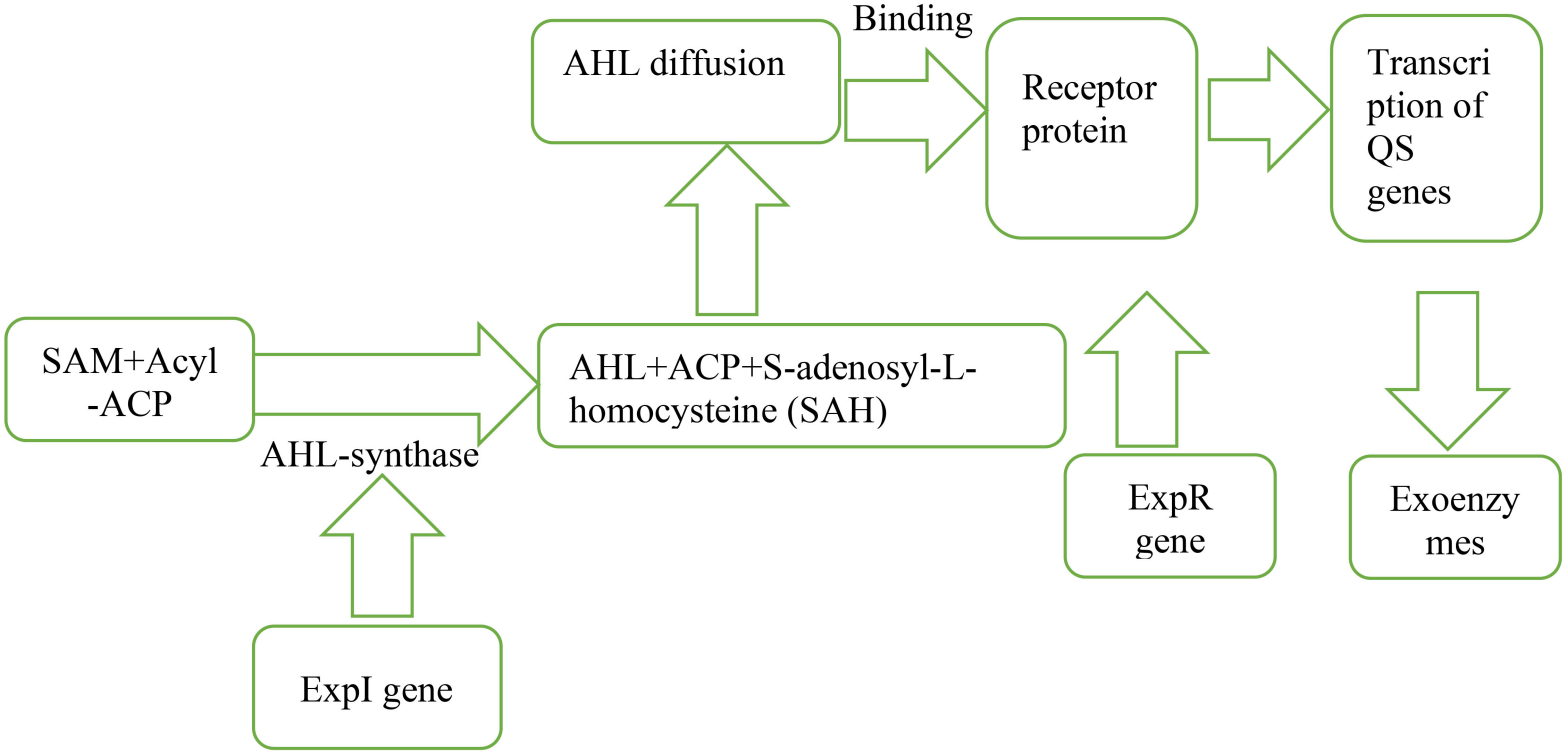
Schematic representation of synthesis of AHL and virulence factors. AHL-acyl-homoserine lactone; SAM- S-adenosylmethionine; Acyl-ACP- Acylated acyl-carrying protein (acyl-acyl carrier protein); SAH-S-adenosyl-L-homocysteine; ExpR – transcriptional regulator; ExpI- AHL synthase.

Some information about mechanisms by which soft rot bacteria impact plants, and conditions for the development of these pathogens are described in the review of authors (Charkowski, 2018), an understanding of which is necessary for comprehensive control measures that target not only the pathogens themselves but also the conditions that facilitate their proliferation. Mechanisms of virulence expression are also very well described in some works (Põllumaa et al., 2012; Sibanda et al., 2018; Vesuna and Nerurkar, 2020; Prazdnova et al., 2022). Synthesis of AHL and virulence factors is shown in **Figure 1**.

The mechanism of virulence expression includes the synthesis of AHL molecules by the enzyme AHL synthase, which is the product of the ExpI gene (Põllumaa et al., 2012; Sibanda et al., 2018; Vesuna and Nerurkar, 2020; Prazdnova et al., 2022).

The synthesis involves substrates S-adenosylmethionine (SAM), the source of the homoserine lactone, and Acylated acyl-carrying protein (acyl-ACP), the source of the acyl chain. Then, after the synthesis of signal molecules, they diffuse into the environment through active or passive transport. As the density of the bacterial population increases, the concentration of AHLs in the environment increases. When the concentration of AHLs reaches a threshold level, they bind to the receptor protein encoded by the ExpR gene. This receptor protein activates the transcription of QS-regulated genes. Thus, the binding of AHLs to ExpR triggers the expression of genes responsible for virulence factors, including exoenzymes. This explanation highlights how quorum sensing works in *Pcc*, focusing on its molecular mechanisms and its role in soft rot pathogenesis.

Understanding the biology and mechanisms of soft rot pathogens including quorum sensing can help to design effective control methods. The current strategies for mitigating the activity of soft rot, particularly in tomatoes, focus on cultural practices, physical and environmental controls, conventional methods, biological control, and some advanced methods such as quorum quenching (Garge and Nerurkar, 2017; Gutierrez-Pacheco et al., 2018).

Although QS-regulated production of plant cell wall -degrading enzymes, including pectinases, cellulases, and proteases, is a common feature of soft rot bacteria, its expression and impact on disease development vary significantly among species, particularly in tomato systems. This variability in QS regulation among soft rot bacteria may influence the effectiveness of quorum quenching strategies. The structure of signaling molecules, regulatory pathways, and environmental responsiveness can lead to variable sensitivity of pathogens to QQ agents.

Environmental factors play important role in modulating QS-regulated virulence in tomato. Temperature is a key determinant, with optimal expression of virulence factors and rapid tissue maceration at 25-30 ^0^C. Moisture is equally important, because high humidity facilitates bacterial spread, promotes cell-to-cell communication, and enhances soft rot development.

Despite significant advances in understanding quorum sensing (QS) in soft rot bacteria, several important limitations and resolved questions remain. One major challenge is the variability of QS systems among strains within the same species, this suggest that QQ approaches effective against one strain or species may not be universally applicable. Moreover, the influence of plant signals and microbial community interactions may reduce or enhance the effectiveness of QQ agents under field conditions. Therefore, successful QQ-based interventions will likely require a context-dependent approach, taking into account pathogen diversity, host-specific factors, and environmental conditions. Future research should focus on elucidating QS dynamics in complex plant environments and on developing broad-spectrum or adaptable QQ-strategies.

Recent studies (Fei et al., 2026) on *Pectobacterium carotovorum subsp.brasiliense*, a major tomato soft rot pathogen, highlight the mechanisms of virulence regulation, but reveal important gaps, for example although transcriptomic analysis have identified of multiple pathways such as phosphotransferase systems, two-component regulatory systems, secretion, and chemotaxis pathways, but the role of quorum sensing (QS) in coordinating these processes is not explicitly addressed in many studies, and its integration with global regulatory networks remains insufficiently understood. Furthermore, most studies focus on individual strains under controlled conditions, limiting our understanding of strain-level variability and the behavior of these systems in complex plant environments.

Studies (Zhang et al., 2026a) using antagonistic bacteria such as *Bacillus amyloliquefaciens* to mitigate *Pectobacterium carotovorum subsp. brasiliense* showed results demonstrated suppression of Pcb through competition for space and nutrients, as well as by the induction of plant defense responses, with emphasis on transcriptome-wide gene expressions alterations involved in plant-pathogen interactions, and the activation of MAPK (Mitogen-Activated Protein Kinase) signaling pathways, elevated defensive enzyme activities (polyphenol oxidase and phenylalanine ammonia-lyase), modulation of hormone signaling, and accumulation of secondary metabolites (total phenols, flavonoids and lignin) in tomato fruits. However, the role of quorum sensing (QS) in these interactions remains largely unexplored, and it is unclear whether biocontrol agents interfere with QS-regulated virulence of Pectobacterium spp., for example through signal degradation or modulation of bacterial communication. Moreover, important gaps remain, particularly given that biocontrol agents actively reshape the plant-associated microbiome, which may indirectly influence QS dynamics and pathogen behavior. These uncertainties complicate the design of quorum quenching strategies, as effectiveness of QQ may depend not only on pathogen-specific QS systems but also on interactions with host responses and microbial communities.

Compared with studies focusing primarily on pathogen virulence or host defense responses (Fei et al., 2026; Zhang et al., 2026a) work of authors (Xie et al., 2026) on quorum quenching strategies provides more direct insights into the role of quorum sensing (QS) in soft rot pathogenesis, particularly, in *Pectobacterium carotovorum subsp. carotovorum.* For example, AHL-degrading cell-free lysates derived from *Bacillus thuringiensis* have been shown to significantly reduce QS signal molecules and suppress QS-regulated virulence traits, including extracellular enzyme production, motility, and biofilm formation, without affecting bacterial growth. However, despite these advances, several important limitations remain. Most studies focus on single pathogen species or strains (for example, in this work *Pectobacterium carotovorum subsp. carotovorum)*, and the variability of QS systems across *Pectobacterium* and *Dickeya* species is not fully addressed. In addition, the potential impact of QQ agents on the broader plant-associated microbiome remains largely unexplored, raising concerns about unintended ecological effects. Furthermore, the interaction between plant-derived signals and bacterial QS systems is rarely considered, despite evidence that host responses may influence pathogen behavior.

Thus, these gaps are important for the development of QQ-based control strategies, as their effectiveness may depend on pathogen diversity, environmental conditions, and complex host-microbe interactions.

## 3 Current strategies for mitigation

A wide range of strategies and approaches to mitigate tomatoes’ soft rot differ in their mechanisms of action, efficacy, and practical applicability. **Table 3** summarizes the main approaches, highlighting disruption or prevention of the development of bacteria-causing disease, application stages, advantages, and limitations.

**Table 3:** Comparative analysis of strategies to mitigate tomato soft rot: mechanisms, application stages, efficacy, and limitations.

Category	Strategy/approach	Mechanism	Application stage	Efficacy	Advantages	Limitations	References
Physical and Environmental Control	Temperature and humidity management	Suppression of pathogen growth and metabolic activity through controlled low storage temperature and reduced humidity conditions	Postharvest, preharvest (greenhouse)	Moderate to high	Simple, widely applicable, non-chemical	Limited efficacy alone, and in warm climate, energy cost	(Alippi et al., 1997; Bartz et al., 2012; Bartz et al., 2020; Ranjan and Singh, 2020)
	Agronomic, Sanitation and hygienic practices	Regular cleaning, crop rotation, removal of infected plant debris, soil drainage, proper water and fertilizers management, prevention of mechanical damage	Preharvest and postharvest	Moderate to high (indirect disease suppression)	Prevents contamination; improves yield and plant health; enhances beneficial microbiomes	Indirect effect; Requires consistent implementation and precise management, climate-dependent	(de Castro and Schjoerring, 2024; Liu et al., 2024; Niu et al., 2024).
Chemical control	Copper-based antimicrobials	Disruption of bacterial cell membranes and enzyme systems via copper ion toxicity	Primarily preharvest	Dependent on conditions	Broad -spectrum activity, rapid contact action, multi-target mechanism	Accumulation, environmental impact, regulatory restrictions	(La Torre et al., 2018)
	Antimicrobial compound (primarily bacteriostatic) potassium tetraborate tetrahydrate (B4K2O7.4H2O) (PTB)	Disruption of microbial metabolism and inhibition of enzyme activity	Postharvest	Moderate	Low toxicity	Limited spectrum; variable efficacy	(Çelikezen et al., 2014; Ahmed et al., 2017a)
	Chlorine-based disinfectants (chlorine dioxide)	Oxidative inactivation of bacterial cells (membrane and enzyme damage)	Postharvest t	Moderate to high (surface decontamination)	Rapid action, widely used	Safety concerns	(Mahovic et al., 2007).
	Organic acids (e.g., kojic acid; Ferulic acid-transferable concept)	Disruption of membrane integrity, induction of host defense, ROS -induced oxidative stress, membrane lipid peroxidation, DNA interaction	Postharvest	High (*in vivo*, and *in vitro*)	Natural origin; low toxicity; effective control; minimal impact on fruit quality, enhances host resistance	Limited field validation; concentration-dependent effects	(Xin et al., 2025; Luo et al., 2026)
	Antibiotics (usually as benchmark-streptomycin, oxytetracycline)	Inhibition of protein synthesis in bacteria	Postharvest (experimental)	High (positive control)	Strong antibacterial effect	Resistance development, restrictions, environmental concerns	(Vu et al., 2022)
Biological control	Antagonistic microorganisms	Competitive exclusion, production of antimicrobial compounds, and induction of plant defense responses (activation of MAPK signaling, hormone pathways, secondary metabolite accumulation; activation of antioxidant and ROS-scavering systems)	Preharvest and postharvest	Moderate to high (variable depending on strain and conditions)	Environmentally friendly, multifunctional action; enhances host resistance; reduces chemical use	Variable efficacy, environmental conditions, strain-specific performan-ce	(Raoul des Essarts et al., 2016; Singh et al., 2021; Zhang et al., 2024)
Advanced/targeted strategies	Quorum quenching	Disrupting bacterial communication to prevent coordinated expression of virulence factors (Enzymatic degradation of AHL (using lactonases/acylases) and/or synthetic AHL analogs that block receptor activation (e.g., ExpR).).	Preharvest, postharvest	Moderate to high (in controlled conditions)	Reduced resistance, targeted approach	QS variability, limited field validation, stability of enzymes	(Fernandes et al., 2010; Singh et al., 2021; Alghamdi et al., 2023)
	Antimicrobial peptides/endolysins	Direct disruption of bacterial membranes and cell walls	Mainly postharvest; preharvest	Rapid effect	Strong antibacterial activity, broad spectrum	Stability and non-cost, side effects	(Nam and Kim, 2026)
Integrated approaches	Combining Multiple Strategies	Synergetic action of physical, chemical, and biological methods to suppress pathogen growth and virulence, and to enhance control	Preharvest and postharvest	High because of synergistic effects	Reduced reliance on chemicals, improved efficacy	Complexity of implementation, cost	(Liu et al., 2024)
	Nanoparticle-based formulations (including nano-mediated QQ approaches)	Nanoparticles-mediated enhanced antimicrobial activity via membrane disruption, oxidative stress, metabolic inhibition; cellular damage; modulation of endophytic microbiome and associated metabolites (e.g., biosynthesized silver nanoparticles)	Preharvest and postharvest (experimental)	Moderate to high	Strong antibacterial activity against *Pectobacterium carotovorum;* green synthesis; microbiome preservation	Nanoparticles accumulation, potential toxicity, lack of field validation, regulatory challenges	(Fernandes et al., 2010; Alghamdi et al., 2023; Beltrán Pineda et al., 2024; Gautam et al., 2024; Xie et al., 2026)
	Microencapsulation or biopolymer-coating solutions	Controlled release of antimicrobial or QQ agents; protection of active compounds	Mainly postharvest, emerging preharvest use	Moderate to high	Improves stability, sustained release, targeted delivery	Scale-up challenges, Formulation complexity	(Saberi Riseh et al., 2023; Kolomiiets et al., 2024)

Agronomic practices such as irrigation and nitrogen management refers to preharvest and can significantly alter rhizosphere microbiome composition, potentially influencing pathogen establishment indirectly (Niu et al., 2024).

Over the last few decades, chemical control strategies have been applied to control plant diseases. However, the overuse and repeated use of several chemical pesticides, such as antibiotics and copper compounds, has caused pollution of the soil environment, a decrease in the population of beneficial microorganisms, residual toxicity on food commodities, and the occurrence of bactericide-resistant strains. Another challenge caused by the overuse of conventional methods are resistance of pathogenic microorganisms to chemicals.

In recent years, consumer demands have tended towards food that contains lower levels of chemical preservatives (or antimicrobials) and exhibits more fresh-like and natural characteristics (Vilaplana et al., 2020). This fact has forced researchers and the industry to develop new food alternative strategies. Therefore, there is an increased need for technologies that can safely and effectively inhibit microorganisms’ growth, reduce postharvest losses, and extend the market quality in products”. Nowadays, using conventional methods to prevent this disease causes new challenges, such as pesticide resistance and environmental pollution (Wang et al., 2017). That’s why biological control strategies are highlighted as environmentally friendly approaches in comparison with conventional methods (Abd-El-Khair et al., 2021).

**Table 3** shows that comparative evaluation of these mitigation strategies reveals significant differences in efficacy, applicability, and practical constraints.

Physical and environmental controls, including temperature and humidity management, remain essential preventive measures.

From literature follows that soft rot caused by *Pectobacterium carotovorum* is strongly influenced by temperature, with significantly higher tissue maceration observed at elevated temperatures (e.g., >80% rot at 35 °C compared to ~27% at 21 °C), highlighting the importance of temperature management during storage (Rehman et al., 2022). Although these findings were derived from potato, *Pectobacterium carotovorum subsp.carotovorum* infects a wide range of crops, including tomato; thus, the strong temperature dependence of disease development highlights the importance of low-temperature management as a key strategy for mitigating soft rot.

Disease observations in greenhouse-grown tomato plants also indicate that soft rot incidence is higher under conditions of elevated humidity, particular in wetter environments (Alippi et al., 1997). In combination with temperature-dependent disease progression, these findings confirm that environmental factors play a critical role in the epidemiology of *Pectobacterium carotovorum*, and management of temperature and humidity should be regarded as a key component of strategies aimed at mitigating soft rot in tomato systems, and although these methods are less effective as other treatments, they play a critical role in reducing pathogen proliferation and disease development, especially during postharvest storage.

Chemical treatments, such as chlorine dioxide and antibiotics, generally provide rapid and high antibacterial efficacy; however, their use in increasingly limited by regulatory restrictions, residue concerns, and environmental impacts. Copper-based antimicrobials, although broad-spectrum, face similar limitations due to accumulation in soils and tightening regulations.

In contrast, biological control strategies, including antagonistic microorganisms such as *Bacillus* spp., and plant-growth promoting bacteria (PGPB), offer environmentally friendly alternatives. These approaches often combine direct antagonism with induction of host resistance, but their efficacy can be variable under field conditions and depends on formulation stability and environmental factors.

Advanced targeted approaches, such as quorum quenching (QQ), antimicrobial peptides, and nanoparticle-based systems, represent emerging solutions. QQ strategies target bacterial communication and virulence rather than growth, reducing selective pressure for resistance; however, their practical application is constrained by challenges related to delivery, stability, and scalability. Similarly, nanoparticle-based treatments demonstrate high antimicrobial efficacy under experimental conditions but raise concerns regarding toxicity, regulatory approval, and environmental safety.

It is important to note that boundaries between different control strategies are often not clearly defined. For example, certain biological agents exert their effects not only through direct antagonism but also by interfering with quorum sensing systems, thereby combining biological control and anti-virulence mechanisms. For example, certain Bacillus spp. have been shown to suppress virulence of *Pectobacterium* by inhibiting QS signaling pathways, thereby combining biological control and QSI-based mechanisms within a single approach (Singh et al., 2021). Some studies highlight that nanoparticles can act as both antimicrobial agents and enhancers of quorum quenching, artificial modulation of QS via engineered nanostructures, and therefore are discussed in both contexts (Fernandes et al., 2010; Alghamdi et al., 2023). Although most quorum quenching studies in plant pathology remain at the experimental stage (Rehman et al., 2022), advances in other fields demonstrate that nanocarrier systems can significantly enhance the delivery, stability, and anti-virulence efficacy of QS inhibitors (Nafee et al., 2014). However, effective and sustainable strategies for controlling Pectobacterium spp., pathogen for soft rot disease, are still a challenge for scientific research, highlighting the need for alternative approaches, especially within the One Health framework (Nam and Kim, 2026).

That’s why in practical disease management, these strategies are not applied independently but are integrated within comprehensive disease management programs. Such integration of preventive, biological, and targeted approaches reflect the principles of integrated pest management (IPM) and provides a more sustainable and effective framework for controlling tomato soft rot.

## 4 Quorum quenching strategy

Traditional methods of control often involve the utilization of chemical pesticides, which adversely affect human health and the environment. Over recent years, there has been an increasing interest in alternative biocontrol methods for the management of field and storage diseases.

One of the promising alternatives is the quorum quenching method that destroys the communication systems for producing enzyme pectinase by virulence microorganisms (Singh et al., 2021; Rehman et al., 2022).

**Figure 2:**
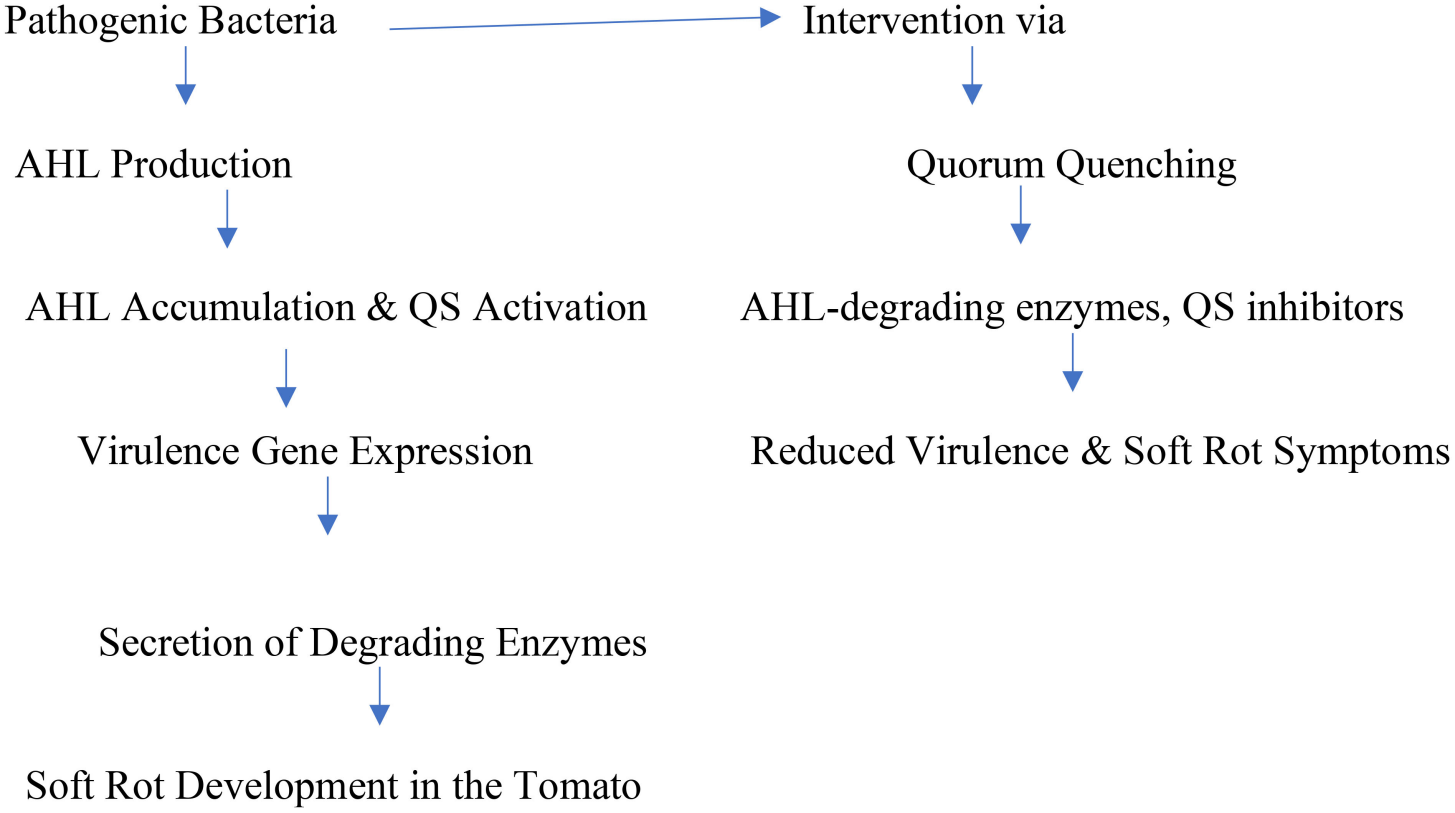
Mechanism of QS and intervention via QQ.

The knowledge of the mechanism of QS allowed us to create strategies for QQ, as shown below in the **Figure 2**.

QS signaling relies on autoinducers, such as acyl-homoserine lactones (AHLs) in Gram-negative bacteria and oligopeptides in Gram-positive.

*Quorum sensing is* utilized by several bacterial species to regulate the functioning of the virulence-induction genes to produce the enzyme pectinase, causing destruction of the shell of vegetables and fruits (Waters and Bassler, 2005; Jeyakumar et al., 2024). QQ interferes with QS by either degrading signaling molecules, blocking their receptors, or otherwise preventing signal transmission (Singh et al., 2021). This disruption stops bacteria from coordinating their pathogenic behaviors, making QS an attractive target for sustainable disease management strategies.

The advantages compared with the use of conventional pesticides is that quorum quenching (QQ) does not impose direct selective pressure on bacterial populations, bacteria developing resistance to QQ is significantly lower (Zhu et al., 2023).

The main points illustrating the QQ microorganisms and how they compete with or disrupt the AHL-based quorum sensing are shown in the table below.

To reflect the QQ strategies in plant systems, **Table 4** distinguishes between different levels of evidence, including *in vitro*, in planta, and postharvest studies, and highlights their relevance to soft rot pathogens, and tomato crops.

**Table 4:** Quorum quenching strategies: mechanisms, evidence level, biological outcomes, and relevance to tomato.

Mechanism category	Examples of QQ agent/references	Target pathogen/host	Mechanism of action	Evidence level	Biological outcome	Relevance to tomato
Enzymatic QQ (QS signal degradation)	AHL lactonases- LrsL lactonase (metallo-β-lactamase superfamily QQ enzyme from the genus *Labrenzia* sp.*) (*Salem and Abd El-Shafea, 2018*).*	Model QS system Pseudomonas aeruginosa	degrade AHL, disrupting QS	*In vitro*	Disrupting AHL signaling, biofilm formation, exopolysac-charide production	Indirect (AHL-based QS conserved in soft rot pathogens), and enzyme can be used in plant protection
	*Bacillus thuringiensis* KMCL07 cell-free lysate (Mahovic et al., 2007)	*Pectobacterium carotovorum subsp. carotovorum*	QQ activity due to the presence of the AiiA lactonase KMMI17 encoded by the aiiA gene	*In vitro*	Reduction of AHL production, PCDWE, biofilm formation and motility, and decreased tissue maceration	same soft rot pathogen affecting tomato
	*Lysinibacillus* sp. Gs50 (Garge and Nerurkar, 2016)	*Pectobacterium carotovorum* subsp. *carotovourm* BR1 *That produces* 3-oxo-hexanoyl homoserine lactone (3OC6HSL)	AHL laconase (AdeH)-mediated degradation of AHL signals (3OC6HSL) via lactone ring hydrolysis	*In vitro+in planta* (potato, carrot, cucumber)	Reduction of AHL signals, attenuation of soft rot symptoms	Same soft rot pathogen affecting tomato
	*Bacillus* spp (Garge and Nerurkar, 2017).	*Pectobacterium carotovorum* subsp. *carotovorum*	AHL degradation leading to Reduction in virulence determinant enzyme production	*In vitro + in planta* (in mung bean)	Reduction of virulence enzyme production, attenuation of soft rot symptoms	Same soft rot pathogen affecting tomato
Signal inhibition (QSI, non-enzymatic)	Bacillus sp. OA10 *Extract* (Singh et al., 2021)	Pectobacterium carotovorum subsp. carotovorum/ potato tissue	inhibition AHL synthesis in Pcc, and disruption of the two QS pathways ExpI/ExpR and CarI/CarR in Pcc	*In vitro+in planta*	Inhibition of plant cell wall degrading enzymes (PCWDE) and carbapenem production	same soft rot pathogen affecting tomato
	AHL-degrading *Bacillus* spp. (isolates As30, Gs42, Gs52) (Garge and Nerurkar, 2017)	Pectobacterium carotovorum subsp. carotovorum/potato, carrot, cucumber, mung bean	Enzymatic degradation of AHL molecules (lactonase activity) disrupting QS-regulated virulence	*In vitro+in planta* (multiple host modes)	Decreasing production of virulence enzymes, attenuation of soft rot	same soft rot pathogen affecting tomato
	*Ochrobactrum* sp. A44, *Rhodococcus, Pseudomonas, Bacillus, and Delftia* (Jafra et al., 2006)	Pectobacterium carotovorum subsp. Carotovorum/potato tubesr	Intracellular degradation of AHL molecules (non-lactonase mechanism), leading to disruption of QS-related virulence	*In vitro+in planta*	Decreasing: AHL signaling, pectinolytic enzyme production, tissue maceration	same soft rot pathogen affecting tomato
	*Probiotic bacteria Lactobacillus acidophilus* strain 30SC (Kim et al., 2018)	*Escherichia coli*/swine gut microbiota	Inhibition of AI-2 quorum sensing signals without affecting bacterial growth	*In vitro+in vivo* (in animal model)	Decreasing QS activity, modulation of microbial community	Perspective for plant systems
	Plant-derived compounds, phytochemicals (Nazzaro et al., 2013; Joshi et al., 2015; Joshi et al., 2016; Zhang et al., 2023) (phenolic compounds (Joshi et al., 2016), essential oils such as oregano (Joshi et al., 2016; Zhang et al., 2023), and clove oil (Joshi et al., 2016; Zhang et al., 2023)	subsp. *carotovorum* (Joshi et al., 2015; Zhang et al., 2023), Pectobacterium carotovorum subsp. Brasiliense (Joshi et al., 2015; Joshi et al., 2016), ectobacterium. aroidearum (Joshi et al., 2015; Joshi et al., 2016), Pectobacterium atrosepticum (Nedaienia and Fassihiani, 2011) */postharvest* (Zhang et al., 2023)	Antibacterial activity combined with inhibition of QS regulated virulence traits (Joshi et al., 2015; Zhang et al., 2023) Inhibition QS signaling via interaction with ExpI/ExpR, and suppression of QS gene expression (Feng et al., 2024)	*In vitro+in plnta* postharvest	Reduced virulence factor expression and biofilm formation (Joshi et al., 2016) inhibiting the *Pcc* virulence-associated factors such as activity, motility, toxicity, biofilm formation, and enzyme secretion to various degrees (Joshi et al., 2015; Zhang et al., 2023)	Direct (soft rot pathogen)
Receptor blocking (signal perception inhibition)	Small-molecule quorum-sensing inhibitors (AHL analogs, halogenated furanones)/synthetic and natural compounds (Geske et al., 2005)	Gram-negative bacteria (model QS systems)	Competitive binding to LuxR-type QS receptors, blocking signal perception and QS-regulated gene expression)	*In vitro*	Reduced biofilm formation and attenuation of QS-regulated virulence traits	Indirect (AHL-based QS conserved in *Pectobacterium* and *Dickeya)*
	Natural and synthetic quorum sensing inhibitors (QSIs) (Grandclément et al., 2016)	Broad gram-negative bacteria	*Interference with QS signal perception by binding to receptors or disrupting signa-receptor interaction*	Conceptual/literature-based	Inhibition of WS-regulated gene expression, reduced virulence and biofilm formation	Indirect (QS conserved in soft rot pathogens)
Multifunctional/including indirect or QQ-related effect systems	Pseudomonas putida PA14H7 and Pseudomonas fluorescens (Raoul des Essarts et al., 2016)	Pectobacterium atrosepticum Dickeya dianthicola Pectobacterium carotovorum subsp. carotovorum Dickeya solani/potato plants and tubers	May produce antagonistic or antimicrobial compounds, compete for nutrients and space, possible indirect QS interference	*In vitro+in planta* (green-house)	Blackleg severity, pathogen transmission to tuber progeny, soft rot symptoms	Relevant soft rot pathogens
	*Bacillus amyloliquefaciens* (strains AB7, AB21, and AB30) (Prazdnova et al., 2022)	Pectobacterium carotovorum subsp. brasiliense/tomato fruits/postharvest	Competitive colonization (biofilm formation), nutrient and space competition, induction of host responses, activation of defense-related genes and secondary metabolite accumulation	*Postharvest(in planta, transcriptomic analysis)*	*Decreasing soft rot incidence, host resistance, enhance antioxidant and defense responses*	Tomato soft rot pathogens
	*Bacillus amyloliquefaciens* (Zhang et al., 2024)	Pectobacterium carotovorum subsp. brasiliense/tomato fruits/postharvest	Antibiosis via lipopeptides (C12-C15 surfactin analogues, along with C14-C16 iturins and C16-C17 fengycins); induction of host defense through enhanced ROS scavenging systems, including several antioxidases and the enzymes related to glutathione peroxidase (GPX) and ascorbate-glutathione (AsA-GSH) cycles, and non-enzymatic system for ROS scavenging (AsA, GSH)	*In vitro+postharvest (in planta)*	Reduced soft rot symptoms, enhance antioxidant defense, decreased ROS accumulation (H2O2 and malondialdehyde (MDA) levels were reduced)	Tomato soft rot pathogens
	Bacillus subtilis, Pseudomonas fluorescence, Pseudomonas aeruginosa, and Streptomyces spp (Salem and Abd El-Shafea, 2018).	Erwinia carotovora subsp. carotovora (Pectobacterium carotovora subsp. carotovora)/potato tubers	Antibiosis (antimicrobial compounds, enzymes), competition, induced resistance (ISR), possible indirect QS interference	*In vitro+in planta*	Decreasing infection rate, soft rot symptoms	Relevant soft rot pathogens
	*Bacillus subtilis, Bacillus pumilus, Bacillus megaterium, Pseudomonas fluorescens, Trichoderma harzianum, Trichoderma viride* and *Trichoderma viren*s (Kolomiiets et al., 2024)	*Pectobacterium. carotovorum* *subsp. carotovorum*/vegetables	Antibiosis (antimicrobial compounds, siderophores), competition, possible induced resistance, possible indirect QS interference	*In vitro+in planta+storage (postharvest)*	Decreasing infection rate, soft rot symptoms (from up to 83%), tissue maceration, prolonged storage	same soft rot pathogen affecting tomato
	*Bacillus amyloliquefaciens* strain Ar10 (Azaiez et al., 2018) (producing glycolipid -like compounds)	*Pectobacterium carotovorum* strain/potato tubers	Membrane disruption (glycolipid surfactant), leading to cell lysis	*In vitro+postharvest*	Reducing severity of disease symptoms, killing rate to 96%	same soft rot pathogen affecting tomato
	Streptomyces diastatochromogenes strain sk-6, and Streptomyces graminearuss strain sk-2 (Doolotkeldieva et al., 2016)	Pectobacterium carptovorum (formely Erwinia carotovora sp. Carotovora)/potato tubers	Antibiosis via production of antimicrobial metabolites (e.g., polyketides)	*In vitro +storage (postharvest)*	Decreasing soft rot symptoms, prolonged storage stability	same soft rot pathogen affecting tomato
	gamma-caprolactone (GCL), 6-caprolactone (6CL) and 4-heptanolide (HTN) (Cirou et al., 2007)	Pectobacterium atrosepticum/potato rhizosphere	Stimulation of NAHL-degrading (QQ) bacterial populations in the rhizosphere, altering microbial community structure	*In vitro+in planta (rhizosphere, tuber assay)*	Enhanced AHL degradation, reduced soft rot symptoms	Related soft rot pathogen affecting tomato
	Fungi: *Trichoderma* spp (Mukherjee et al., 2012; Alexander et al., 2014; Haryadi et al., 2019; Alexander et al., 2021*)*.		Fungal species produce antimicrobial substances that inhibit AHL-producing bacteria.	Conceptual/literature- based	Reduced pathogen colonization and disease severity	Potential for suppression of soft rot pathogen

One of the most promising and advanced strategies of inhibition is biological inhibitors, which use certain microorganisms that secrete enzymes that destroy QS signaling pathways, including inhibition of AHL synthesis. These include bacteria of the genus *Bacillus*, fungi of the genus *Penicillium* and others (Koh et al., 2012; Lim et al., 2019). The most common bacterial strains, including soil bacilli capable of inhibiting biofilm formation, can be found in the rhizosphere of plants, various bodies of water, and other microbial communities. Mainly genera *Agrobacterium*, *Bacillus*, *Pseudomonas*, *Delftia, Ochrobactrum*, and *Rhodococcus, Burkholderia*, *Streptomyces*, and *Trichoderma* are very well-known for their antimicrobial capability and synthesis of a wide range of bioactive compounds (Ma et al., 2007; Alexander et al., 2014; Azaiez et al., 2018; Charkowski, 2018; Haryadi et al., 2019; Alexander et al., 2021) and abilities to degrade AHL.

Authors (March and Bentley, 2004; Geske et al., 2005; Harsimran Kaur and Harsh, 2014; Grandclément et al., 2016; Alexander et al., 2021; Raman et al., 2022; Ayaz et al., 2023; Saberi Riseh et al., 2023; Zhu et al., 2023; Jeyakumar et al., 2024; Kolomiiets et al., 2024; Luo et al., 2026) cover traditional and biological control approaches, including promising methods like quorum quenching, highlighting the challenges of pathogen resistance and the need for sustainable management strategies.

The biocontrol effect of *Bacillus subtilis* on postharvest soft rot was determined to trigger ROS outbreaks, which induced the defense response of the fruits (Feng et al., 2024).

Compared with enzymatic quorum quenching approaches, receptor-blocking strategies targeting LuxR/ExpR-type receptors remain poorly explored in soft rot pathogens of tomato, despite their promising anti-virulence potential. Most receptor-blocking studies have been performed in model bacterial systems, whereas their application to plant pathogens such as *Pectobacterium* spp. remains poorly explored.

Recent comprehensive reviews have provided detailed insights into quorum sensing and quorum quenching mechanisms, including the diversity of signaling molecules (e.g., AHLs, AI-2, DSF) and the wide range of QQ strategies such as signal degradation, enzymatic inactivation, and receptor-level interference (Zhu et al., 2023). These studies offer a valuable theoretical framework and demonstrate the broad applicability of QQ approaches across medical, environmental, and agricultural systems. These reviews have also described application of quorum quenching in soft rot pathogens and use of biocontrol strains. Although the growing interest in quorum quenching strategies, several limitations remain, and these studies also highlight important details, particularly the poor stability of QQ agents and the lack of effective delivery systems for practical applications.

Thus, most studies as described in **Table 4** have been conducted under *in vitro* or controlled conditions, and the stability and persistence of QQ agents under field of postharvest environments are still poorly understood. In addition, potential impacts on non-target microbial communities and the possibility of adaptive responses in pathogens require further investigation. Therefore, while QQ represents a promising anti-virulence approach, its large-scale application in crop protection limited by poor stability and lack of delivery systems and have to be fully validated.

In addition, recent review by (Perfileva et al., 2025) provides a comprehensive overview of *Pectobacterium carotovorum* biology and existing control methods; however, it mainly offers descriptive summaries of individual strategies without integrating them into a coherent application framework. In contrast, the present review provides a focused and integrative perspective by combining quorum quenching, lactic acid bacteria, and microencapsyulation approaches, with particular emphasis on their application in tomato soft rot management, while also drawing on existing knowledge from other crops affected by *Pectobacterium* spp. and *Dickeya* spp (Perfileva et al., 2025).

Thus, to address these critical limitations, this review focuses on LAB-based systems as a promising quorum quenching agents. Lactic acid bacteria (LAB) have attracted increasing attention due to their ability to interfere with quorum sensing systems, as well as their well-established safety, ecological compatibility, and applicability in both food and agricultural systems. Importantly, LAB has GRAS (Generally Recognized as Safe) status by the Food and Drug Administration, which supports their safe use for human and animal consumption (Sadiq et al., 2019; Chen et al., 2023). This makes LAB attractive for commercial development (Sadiq et al., 2019; Chen et al., 2023) including applications in postharvest treatments and edible coatings.

Lactic acid bacteria (LAB) have been described as a promising antagonists for the control of soft rot diseases (Burkholder et al., 1953), and their biocontrol potential attributed to well-established mechanisms such as acidification, production of antimicrobial metabolites (e.g., bacteriocins), and competitive exclusion (Tsuda et al., 2016; Daranas et al., 2019; Qi et al., 2021). In addition to their antimicrobial activity, LAB have also been reported to promote plant growth, distinguishing them from classical quorum quenching-associated genera such as *Pseudomonas*, *Burkholderia*, *Streptomyces*, *Bacillus*, and *Trichoderma* (Jaini et al., 2022). A schematic overview of LAB-mediated plant disease control and growth promotion has been provided by Raman et al. (2022), highlighting their eco-friendly and multifunctional role in crop productivity and soil health. While this framework clearly demonstrates the broad biocontrol potential of LAB, it primarily focuses on classical mechanisms and does not explicitly address their potential role in quorum sensing interference or the challenges associated with their practical application, such as the limited availability of extensive field trials, high application costs, and potential risks related to the transfer of antibiotic resistance genes.

Thus, beyond these established functions, emerging evidence suggests that LAB may also interfere with quorum sensing-regulated processes. For instance (Garge and Nerurkar, 2016; Kim et al., 2018), demonstrated that *Lactobacillus acidophilus* strain 30SC significantly reduced biofilm formation in Escherichia coli O157:H7. Notably, the level of biofilm formation in the presence of LAB cell extracts was comparable to that observed in an AI-2-deficient (l*uxS*) mutant, indicating potential interference with AI-2-mediated quorum sensing signaling.

Although such evidence is primarily derived from non-plant systems, these findings provide a strong basis for extending LAB-mediated quorum quenching strategies to plant pathogen systems, including soft rot caused by *Pectobacterium* and *Dickeya*, which regulate their virulence through both AHL- and AI-2-based quorum sensing systems (Paul et al., 2018).

LAB-derived metabolites, such as unsaturated fatty acids and hydroxylated fatty acids, exhibit antimicrobial activity against a wide range of fungal and bacterial pathogens. In addition, glycolipid biosurfactants produced by LAB contribute to the inhibition of bacterial attachment and disruption of biofilm formation (Patel et al., 2021), processes often associated with quorum sensing-regulated activities (Jaini et al., 2022). These mechanisms collectively support the antagonistic potential of LAB and their role in biocontrol strategies.

While most studies on LAB-mediated suppression of plant pathogens emphasize classical mechanisms such as acidification and bacteriocin production, emerging evidence indicates that LAB may also interfere with quorum sensing pathways. For example (Prazdnova et al., 2022), reported quorum sensing suppression by Gram-positive microorganisms, highlighting potential mechanisms and molecular intermediates involved in signal interference (Vesuna and Nerurkar, 2020). Compared to well-established quorum quenching genera such as *Bacillus* spp., lactobacilli have been less frequently recognized as regulators of pathogen communication and are commonly associated with food fermentation and probiotics applications. Nevertheless, accumulating evidence suggests that LAB may act as potential QQ agents, although their specific role in suppressing QS systems of soft rot pathogens remains insufficiently understood.

In addition to direct antimicrobial effects, LAB have also been explored as sources of bioactive compounds derived from fermented plant substrates. Their food-grade status and metabolic versatility make them attractive candidates for the development of sustainable biocontrol strategies, particularly in postharvest systems (Dopazo et al., 2022; Garmasheva et al., 2024; Tang et al., 2024).

Lactic acid bacteria (LAB) have been investigated in several studies relevant to soft rot pathogens, including direct plant-pathogen systems such as *Pectobacterium carotovorum* (Burkholder et al., 1953; Tang et al., 2025), and broader agricultural or antifungal contexts (Sadiq et al., 2019; Jaini et al., 2022; Jaini et al., 2022; Chen et al., 2023). These studies, primarily based on *in vitro* assays and limited *in vivo* plant models, report antagonistic effects mediated by antimicrobial metabolites, competitive exclusion, and quorum-sensing interference (Pelyuntha et al., 2019).

LAB have also been reported to interfere with biofilm formation, a process often regulated by use of cell-to-cell communication in bacterial pathogens. For instance, *Lactobacillus brevis* and other LAB strains have demonstrated strong inhibitory effects on biofilm development, while some LAB strains are capable of forming their own biofilms, thereby competitively limiting pathogen colonization (Fujita et al., 2007; Gómez et al., 2016; Kim et al., 2019; Wang et al., 2025). These interactions may contribute to the suppression of virulence-related traits in soft rot pathogens.

Importantly, several studies have reported quorum quenching activity of LAB (Pelyuntha et al., 2019), including the ability to inhibit AI-2-mediated signaling without affecting bacterial growth, suggesting direct interference with cell-to-cell communication (Garge and Nerurkar, 2016; Pelyuntha et al., 2019; Darai and Pelyuntha, 2025).

However, despite these promising findings, studies specifically targeting tomato soft rot under greenhouse or field conditions remain scarce, highlighting a critical gap in the practical application of LAB-based quorum quenching strategies.

In addition, under postharvest and storage conditions, the production of organic acids and other metabolites by LAB may affect sensory properties of treated fruits. Therefore, further studies are required to optimize formulation strategies and to validatie LAB performance under these conditions.

In this context, microencapsulation of LAB and other biocontrol agents represents a promising strategy to enhance their stability and effectiveness in controlling tomato soft rot caused by *Pectobacterium carotovorum* and *Dickeya* spp. Microencapsulation protects LAB under harsh environmental conditions and enables controlled release of viable cells or their bioactive metabolites. One of its key advantages is possibility of sustained delivery, which may prolong antimicrobial activity, reduce application frequency, lower labor costs, and improve overall treatment efficiency.

**Figure 3:**
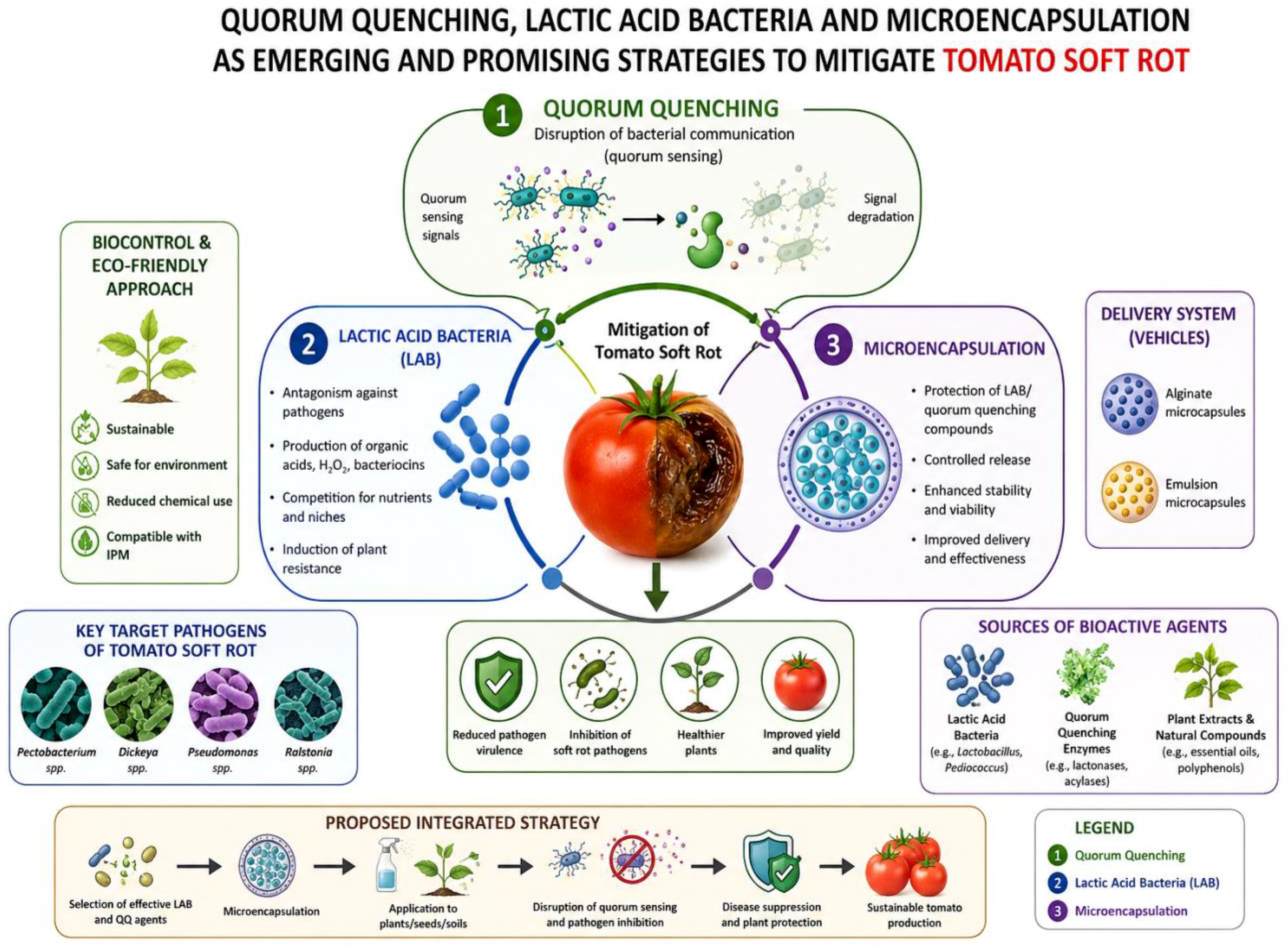
Quorum quenching, lactic acid bacteria and microencapsulation as emerging and promising strategies to mitigate tomato soft rot.

A schematic overview of the integration of quorum quenching, lactic acid bacteria, and microencapsulation as emerging strategies for mitigating tomato soft rot is presented in **Figure 3**.

Evidence for supporting the use of microencapsulated LAB is largely derived from probiotic and food systems (Solanki et al., 2013; Wang et al., 2017; Sun et al., 2023) with some extension to agricultural biocontrol formulations (Saberi Riseh et al., 2023). These studies consistently demonstrate improved viability, environmental tolerance, and controlled release properties. Hovewer, their application to tomato soft rot remains largely inferential, as direct experimental validation in this specific pathosystem is still very limited.

Nevertheless, the available evidence highlights the strong potential of LAB-based systems due to their multifaceted mechanisms of pathogen suppression, including antimicrobial activity, interference with QS-regulated processes, and additional plant-beneficial effects.

## 5 Microencapsulation in agricultural and postharvest systems

Microencapsulation refers to the entrapment of active compounds (core materials) within a protective matrix (wall material), forming particles typically ranging from 1 to 1000 µm. In agricultural and postharvest contexts, this technology is increasingly used to stabilize and control the release of bioactive agents such as essential oils, plant extracts, microbial antagonists, enzymes, and synthetic agrochemicals (Gharsallaoui et al., 2007; Fang and Bhandari, 2010). The primary functional advantages include: a) protection from environmental degradation (light, oxygen, temperature, humidity), b) controlled and sustained release, c) reduced volatility and phytotoxicity, d) enhanced solubility and dispersion and e) improved bioavailability and efficacy. These features are particularly relevant for postharvest systems, where maintaining product quality and reducing microbial spoilage are critical (Burt, 2004; Maurya et al., 2024).

### 5.1 Encapsulation techniques

Microencapsulation methods used in agriculture and postharvest applications can be broadly classified as:

- Physical Methods.
  - Spray drying: Most widely used due to scalability and cost-effectiveness; suitable for heat-stable compounds (Burt, 2004).
  - Freeze drying (lyophilization): Preserves thermolabile compounds but is more expensive.
  - Fluidized bed coating: Enables layering of coatings and controlled release.
- Physicochemical Methods.
  - Coacervation (simple and complex): Uses phase separation of polymers (e.g., gelatin–gum arabic systems).
  - Inclusion complexation: Often involves cyclodextrins for encapsulating hydrophobic molecules.
- Chemical Methods.
  - Interfacial polymerization: Produces well-defined capsules but may involve toxic reagents.
  - *In situ* polymerization: Used for synthetic agrochemical formulations.

The choice of method depends on the nature of the active compound, desired release profile, and application environment (Fang and Bhandari, 2010).

### 5.2 Carrier materials

Carrier materials are critical in determining encapsulation efficiency, release kinetics, and stability. Common classes include:

- Polysaccharides.
  - Chitosan: Antimicrobial, biodegradable, widely used in edible coatings.
  - Alginate: Gel-forming, used for encapsulating microbial agents.
  - Starch and modified starches: Economical and widely available.
- Proteins.
  - Gelatin: Common in coacervation systems.
  - Whey protein isolate: Good emulsifying properties.
- Lipids.
  - Waxes and fatty acids: Used for hydrophobic barriers and slow release.
- Synthetic Polymers.
  - Poly(lactic-co-glycolic acid) (PLGA): Controlled release but limited use in food systems.

Selection criteria include biodegradability, food safety status (GRAS), mechanical properties, and compatibility with the core material (McClements, 2015).

### 5.3 Target applications in agriculture and postharvest systems

#### 5.3.1 Antimicrobial delivery

Encapsulated essential oils (e.g., thymol, eugenol) exhibit enhanced stability and prolonged antimicrobial activity against pathogens such as Botrytis cinerea, Penicillium expansum, and Alternaria spp (Burt, 2004; Maurya et al., 2024).

#### 5.3.2 Controlled release of agrochemicals

Encapsulation reduces leaching, volatilization, and environmental contamination while improving efficacy (Campos et al., 2015).

#### 5.3.3 Biological control agents

Encapsulation protects beneficial microbes (e.g., Bacillus, Trichoderma) from environmental stress and improves shelf life and field performance (Mahato et al., 2021).

#### 5.3.4 Edible coatings and films

Microencapsulated actives incorporated into coatings extend shelf life of fruits and vegetables by reducing respiration rates and microbial growth (Dutta et al., 2011).

#### 5.3.5 Release mechanisms

Release of encapsulated compounds occurs via:

- Diffusion through the matrix.
- Matrix degradation (enzymatic or environmental).
- Swelling and dissolution.
- External triggers (pH, temperature, moisture).

Designing release kinetics is essential for matching application needs, such as sustained antimicrobial activity during storage (McClements, 2015).

In **Table 5** we summarized carriers, target organisms, and outcomes.

**Table 5:** Integrative table: carriers, target organisms, and outcomes.

Carrier material	Encapsulated compound	Target organism/system	Application context	Observed outcome	Reference
Chitosan	Thyme essential oil	*Botrytis cinerea*	Postharvest strawberries	Reduced fungal growth, extended shelf life	(Heidary et al., 2025)
Alginate	*Bacillus subtilis*	Soil-borne pathogens	Crop protection	Improved microbial survival and efficacy	(Saberi-Rise and Moradi-Pour, 2020)
Gum arabic + maltodextrin	Clove oil	*Penicillium expansum*	Apples (postharvest)	Enhanced antifungal activity, reduced decay	(Gharsallaoui et al., 2007)
Cyclodextrin	Eugenol	*Alternaria* spp.	Fresh produce	Increased stability and controlled release	(Fang and Bhandari, 2010)
Lipid-based carriers	Synthetic pesticide	Insect pests	Field application	Reduced volatilization and environmental loss	(Campos et al., 2011)
Whey protein	Carvacrol	Mixed spoilage microbiota	Fresh-cut vegetables	Improved antimicrobial persistence	(Dutta et al., 2009)

### 5.4 Practical considerations for microencapsulated LAB application

Despite promising laboratory results, several practical constraints must be addressed before microencapsulated lactic acid bacteria (LAB) can be widely adopted for tomato soft rot management. Cost and scalability remain key challenges, as encapsulation techniques such as spray-drying, freeze-drying, extrusion, and emulsion-based methods differ substantially in energy demand, throughput, and material costs. Spray-drying is widely regarded as the most industrially scalable and cost-effective approach, although it can reduce cell viability due to thermal stress (Desmond et al., 2002; Fu and Chen, 2011; Huang et al., 2017).

In contrast, freeze-drying provides higher survival rates but involves higher capital and operational costs and is less suited to large-scale agricultural deployment (Nwankwo et al., 2023; Ramachandran et al., 2024; Zhao et al., 2025; Uwineza and Zhang, 2026). Other approaches such as extrusion and fluidized bed coating offer improved cell protection but face limitations in throughput and process standardization (Agriopoulou et al., 2023; Islam et al., 2026).

Compatibility with existing postharvest practices is another critical factor. Encapsulated formulations must be adaptable to standard application methods such as dipping, spraying, or incorporation into edible coatings used in tomato handling systems. Previous studies indicate that microencapsulated microorganisms can be integrated into coating matrices or aqueous suspensions; however, formulation-dependent issues such as particle aggregation, nozzle clogging, and uneven surface distribution may affect application efficiency (Karnwal et al., 2025; Mathura et al., 2025; Pasqualicchio et al., 2026).

Also, shelf life and storage stability are central to commercial viability. Encapsulation is well documented to enhance LAB survival during storage by protecting cells from oxygen, moisture, and temperature fluctuations, thereby extending functional shelf life compared to free cells (Rajam and Subramanian, 2022; Bustamante et al., 2025). However, stability depends strongly on encapsulating materials, residual moisture content, and storage conditions, and long-term performance data under realistic agricultural supply-chain conditions remain limited, particularly for plant disease control applications (Abdulsalam et al., 2025; Davidescu et al., 2025).

## 6 Conclusion and future perspectives

### 6.1 Key messages

- Quorum sensing (QS) is central to the virulence of *Pectobacterium* spp. and *Dickeya* spp., and quorum quenching (QQ) represents a targeted anti-virulence strategy with demonstrated potential to reduce soft rot severity without relying on bactericidal approaches.

- Lactic acid bacteria (LAB) show multifunctional biocontrol activity against soft rot pathogens, including antimicrobial metabolite production, competitive exclusion, and interference with QS systems; however, evidence is still largely limited to *in vitro* and controlled-environment studies.

- Formulation strategies such as microencapsulation and edible coatings can enhance LAB stability and delivery, but their application to tomato soft rot remains insufficiently validated under realistic postharvest and supply-chain conditions.

### 6.2 Specific research gaps

- Identification and validation of LAB strains with quorum quenching activity specifically targeting QS systems in *Pectobacterium* and *Dickeya*, including molecular characterization of signal interference mechanisms.

- Standardized *in vivo* testing in tomato systems, including detached fruit, greenhouse, and field trials, to quantify the efficacy of LAB against soft rot incidence and severity.

- Development of edible coating formulations incorporating LAB, with systematic evaluation of their effects on disease suppression, fruit physicochemical quality, and shelf life during storage.

- Optimization of microencapsulation techniques for agricultural use, including cost-effective production, controlled release profiles, and compatibility with commercial postharvest practices (e.g., dipping and spraying). Recent advances in the technologies that offer promising approaches to mitigate soft rot such as the development of resistant tomato cultivars through conventional breeding and genetic engineering.

- Validation under cold-chain and commercial storage conditions, including assessment of LAB survival, activity, and consistency of disease control performance.

### 6.3 Practical implications for growers and industry

**-** LAB-based treatments could be integrated into existing postharvest workflows, particularly through dipping or coating systems, provided formulation stability and application parameters are optimized.

**-** Microencapsulated formulations may improve product shelf life and ease of handling, but cost–benefit considerations and scalability must be addressed before commercial adoption.

QS-targeted approaches offer a complementary strategy to conventional control methods, potentially reducing reliance on chemical treatments while aligning with increasing regulatory and sustainability demands.
